# The effect of switching costs on choice-inertia and its consequences

**DOI:** 10.1371/journal.pone.0214098

**Published:** 2019-03-25

**Authors:** Nathaniel J. S. Ashby, Kinneret Teodorescu

**Affiliations:** 1 Harrisburg University of Science and Technology, Harrisburg, Pennsylvania, United States of America; 2 Technion–Israel Institute of Technology, Haifa, Israel; University of Sheffield, UNITED KINGDOM

## Abstract

In two studies we provide a novel investigation into the effects of monetary switching costs on choice-inertia (i.e., selection of the same option on consecutive choices). Study 1 employed a static decisions-from-feedback task and found that the introduction of, as well as larger, monetary switching costs led to increases in choice-inertia. While experience and decreases in the similarity of options average payouts (expected value: EV) increased choice-inertia for the option with a higher EV (the EV maximizing option), switching costs increased choice-inertia for the inferior option (the lower EV option): The proportion of total participants showing choice-inertia for the EV maximizing option also increased with switching costs. Study 2 employed a dynamic decisions-from-feedback task where halfway through the task the EV maximizing option became the inferior option. The effect of switching costs increasing choice-inertia for both the EV maximizing and the inferior option was replicated with little impact of the change in options values being detected. In sum, decision makers appear to be sensitive to switching costs, and this sensitivity can bias them towards inferior or superior options, revealing the good and the bad of choice-inertia.

## Introduction

Many of our daily decisions result in repeating our previous choices: Most days we use the toothbrush and toothpaste we used the day before, we drive the same roads to and from work, and buy groceries at the same store. Repeating the same choice over and over, henceforth referred to as choice-inertia, can be the result of an efficient cost-benefit analysis: Once a good option is identified, further exploration of alternative options can lead to a waste of resources (e.g., time, effort, and money). Nevertheless, if the repeatedly selected option is, or becomes over time, inferior to other options, choice-inertia could result in poor outcomes. In real life decisions, evaluating whether choice-inertia is efficient or not is difficult as it is unclear how to quantify the value of repeating one’s last choice and to estimate forgone outcomes (the value of switching to a different option). A cleaner examination of the good and bad of choice-inertia can be obtained in controlled lab experiments where the values associated with each option are better specified.

We start with the observation that many behavioral biases can be described as reflecting situations in which choice-inertia has negative consequences on performance: The status quo effect [1] where individuals prefer the current course of action, the sunk cost effect [2] where decision makers “throw good money after bad”, and default effects [3] where courses of action which are stated as the default are most likely to be pursued. Specifically, once a course of action is initiated, either by the decision maker on previous decisions as is the case with sunk cost and status quo effects, or by external forces as is the case in default effects, carrying out that course of action is observed even if superior (higher value) alternatives exist.

Another set of examples includes the tendency to repeatedly choose suboptimal options while neglecting to explore for the existence of better options, as observed in local maxima stabilization [4] [5] insufficient exploration [6] [7], minimal cognitive investment [8], and the hot stove effect [9]. It is important to note that choice-inertia can lead to poor performance even if the original choice was the best alternative available. This occurs when changes in the environment make the original choice obsolete but the choice strategy does not adapt. Examples of negative consequences of choice-inertia in dynamic environments include learned helplessness studies [10] [11] and the resistance to change literature (e.g. [12] [13]; for review in organizational contexts see [14]).

Despite differing contexts, all the phenomena documented above describe situations in which decision makers stick to a chosen alternative (i.e. exhibit choice-inertia) even when it reduces performance (earnings). Resting on the similarity among these diverse effects we focus on the general tendency to exhibit choice-inertia, with the supposition that all these effects can be placed under a unified umbrella of inertia biases. It is important to note that choice-inertia by itself is not a negative bias [15] [16][17]. As noted, sticking to the best alternative is desired and can be the result of rational cost-benefit analyses (see for example [18], [19]). Nevertheless, negative consequences of choice-inertia could occur in two situations: 1) If initial identification of the superior option was incorrect; 2) If initial identification of the superior option was correct, but the decision maker is unaware of changes in the environment that caused this option to become inferior.

One factor that has been suggested to account for many inertia biases is switching costs. For example, transition costs were referred to as a rational cause for status quo inertia [1]; physical and cognitive costs associated with changing the default were raised as a factor contributing to default effect in organ donations [3]; exploration costs were suggested to be one of the main factors leading to learned helplessness [11]; perceived switching costs was shown to increase user resistance to information systems implementation [12]; and perceived transition costs led to increased reported inertia and decreased acceptance of new technology [13]. However, in each of these studies, switching costs were either speculated to have an effect, or observed rather than manipulated. For example, Dinner et al. [20] measured effort to switch from the default option by asking participants to rate their agreement with the sentence, “I made my choice because it was easier to choose that option”; they also used decision time as a proxy for effort. While ambiguous, their findings suggest that effort will likely have a negative effect when the choice task is difficult, and effort is manipulated. Similarly, Gal [15] argued that inertia biases such as status-quo, endowment effects, and the risky bet premium can be the result of difficult choices that induce ambiguous preferences. Inertia ‘biases’ in this case can occur simply because one does not have enough motivation to make a change. Nevertheless, ambiguity cannot be the sole driving factor behind these effects, given findings that status quo effects are present even when the status quo option is clearly inferior [21].

In contrast, larger switching costs might have a positive effect on expected value (EV) maximization: Selecting options paying higher amounts on average. It might reduce stochastic or random choice, leading to more (desired) consistency. For example, Plonsky et al. [22] showed evidence that in binary choice tasks people search for patterns, even when these do not exist, which can lead to over-switching. Switching cost might therefore reduce the number of suboptimal switches following false beliefs about the dynamism of a static environment (though see [23]). Moreover, switching costs might change the way people acquire information about alternatives. For example, a piecewise sampling strategy where switching is more frequent led to less EV maximization than a comprehensive sampling strategy where one option is sampled repeatedly before switching in one off choices [24]. As such, switching costs could lead to more comprehensive exploration (sampling) strategies, which in turn might increase EV maximization.

In the current paper, we aim to shed light on the influence of switching costs on choice-inertia. Although switching costs are commonly mentioned in the context of inertia biases, to the best of our knowledge, switching costs have yet to be directly manipulated. In addition, the potential positive effects of choice-inertia (e.g. reduced noise, increased consistency) have generally been overlooked. In the current studies switching costs are implemented as an explicit monetary loss follow each switch (change in the option selected on consecutive choices). Following Dinner et al.’s suggestion [20] we also manipulated the difficulty of the task by staggering the degree of similarity (differences/gaps in EV) between the two options. The more similar options are to one another, the more challenging it should be to determine the more advantageous option, and the emergence of ambiguous preferences should become more likely. Preferences which according to Gal [15] should increase choice-inertia. The current simplified setting enables an unhindered look at the consequences of choice-inertia and its development under varying switching costs and similarity conditions.

## Study 1: Inertia in static environments

### Methods

#### Participants

One-hundred and thirty-one (*M*_age_ = 39.22; 47% female) participants were recruited using Amazon Mechanical Turk (MTurk) and received $0.50 plus monetary incentives (*M* = $0.76) based on their decisions (described below). The MTurk platform for on-line participant recruitment mirrors lab-based recruitment in many ways. Crump, McDonnell, & Gureckis (2013) compared lab based and MTurk experiments and found that MTurk experiments led to results similar to those observed in the lab, except learning was slower (e.g. in a category learning task) which they argued is possibly due, “to the more diverse participant sample than is typical in laboratory studies”. An attention check (clicking on an invisible box instead of “continue”) was employed before the study began and participants (N ≈ 147) who failed the check were not allowed to take part. The study was approved by the Technion ethics committee for behavioral studies.

#### Stimuli and procedure

After providing informed consent and answering standard demographic questions (e.g., age and gender) participants were told that they would make 50 choices in several different pairs of options (labeled Option A and Option B). Participants were told that they would receive feedback about the outcome that occurred for the option they picked in points (40 points = $0.01). Three pairs of options were included and were encountered in random order by all participants. Each option paid out a certain outcome and a random amount drawn from a Gaussian (normal) distribution with a mean of 0 points and a standard deviation of 3 points which was added/subtracted from that certain outcome. Therefore, the expected value (EV) of each option was equal to its certain amount. In one pair both options had an EV of 19 points, thus choosing either option returned the same net earnings on average. In the remaining pairs, one option provided a higher average payout than the other: In one pair one option had an EV of 16 points and the other an EV of 22 points (a 6 point difference/gap on average), while in the final pair one option had an EV of 13 points and the other option had an EV of 25 points (a 12 point difference/gap on average). These made up the three EV gap conditions. Participants made 50 consecutive choices in each pair and were informed that they were moving to a new pair of options following their 50^th^ choice from a pair. In addition, participants were randomly assigned to, and informed that they were in, one of three switching cost conditions at the start of the study: 0 (N = 43), 10 (N = 43), or 20 points (N = 45). We informed participants about the switching cost condition they were in to reduce order-effects. For example, if participants had to discover the switching cost condition they were in, the first problem played, particularly in the first few trials, might reflect behaviors/strategies that differed from all others where the switching cost would be known from previous experience. After their first choice in a pair, if a participant in the 10 switching cost condition picked a different option than they had on their previous choice (e.g., they selected Option A on their first choice and Option B on their second choice) they were informed that 10 points had been subtracted from their earnings. Switching cost conditions did not change for a participant during the study. That is, while EV gaps were manipulated *within* participant, switching costs were manipulated *between* participants. Manipulation of switching costs between participants was done to reduce carry over effects, which could occur if switching costs were manipulated within participant. After participants made 50 choices in all three pairs they were informed of their total earnings and thanked for their time.

### Study 1 results

#### The development of choice-inertia with experience (Study 1)

**Fig 1:**
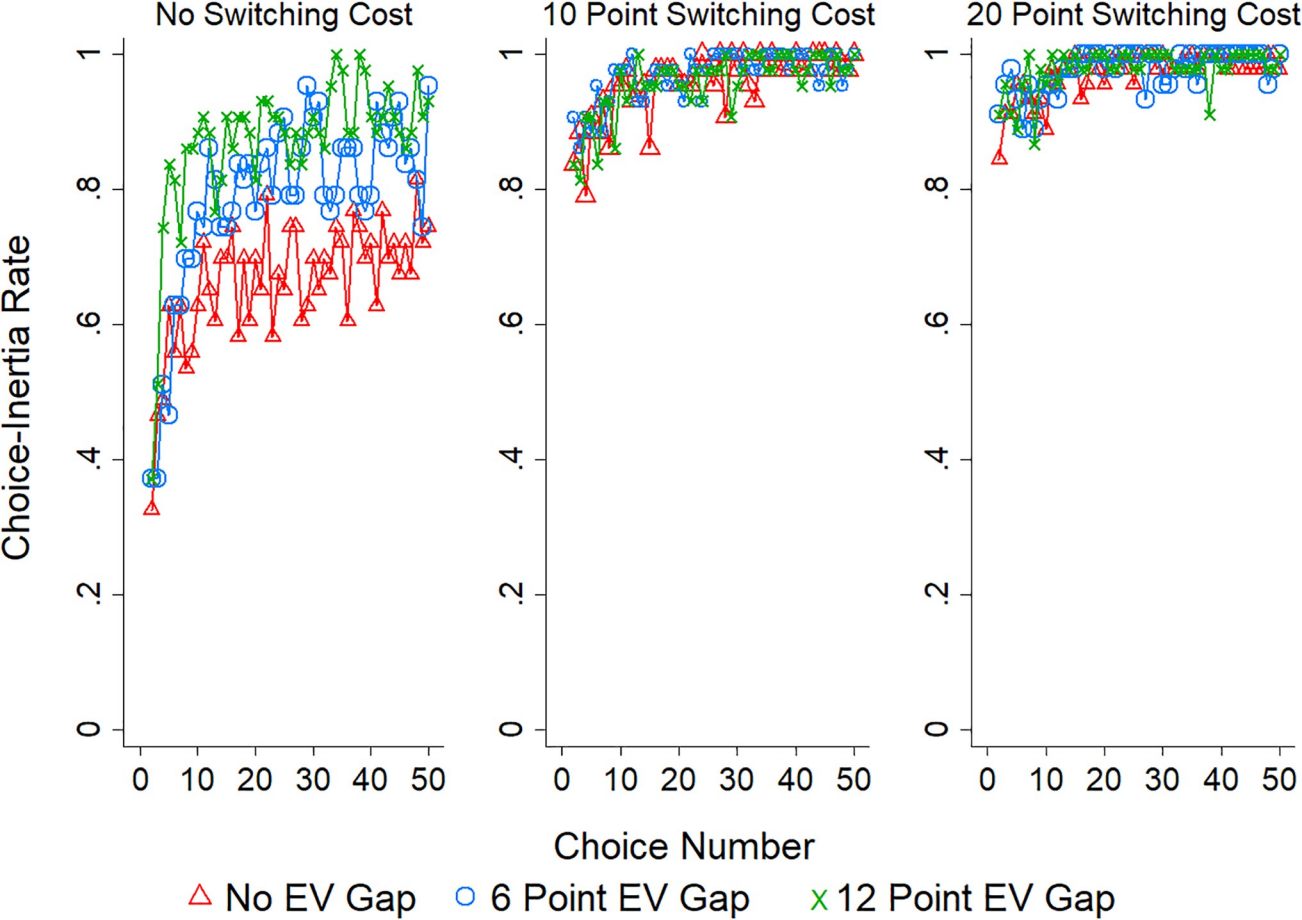
Choice inertia in Study 1. The rate of choice-inertia (choosing the same option on consecutive choices) in Study 1 with separate lines for each EV gap condition (pairs of options with a 0, 6, or 12 point EV gap) and plotted separately by switching cost condition: 0 points (left panel), 10 points (middle panel), or 20 points (right panel).

Fig 1 displays the rate of choice-inertia (choosing the same option on consecutive choices) across the 50 choices with separate lines for each EV gap condition and plotted separately by whether participants were in the 0 (left panel), 10 (middle panel), or 20 (right panel) point switching cost condition. Visual inspection suggests three potential effects: Choice-inertia increased over choices, as the EV gap increased, and as the switching cost increased. See the S1 Appendix for a related investigation of decision times.

To empirically test these observations we performed a logistic regression predicting choice-inertia by choice number, EV gap, switching cost, as well as their interactions: All variables in Study 1 and 2 are entered linearly from smallest to largest and mean centered and repeated measurement is accounted for by clustering on the level of participant [25]. Full mixed effect models (i.e., models with all relevant fixed effects and interactions free to vary within participant) could not be fit (perhaps due to lack of variation in participants choices) reducing our faith in the robustness of the observed effects [26], thus our use of a more conservative method (i.e., cluster correction). The results of this analysis are reported in Table 1. In line with our observations we find the likelihood of choice-inertia increased over choices and as switching costs increased. Interestingly the size of the EV gap between options did not significantly affect choice-inertia. The interaction between choice number and cost condition indicated that the positive effect of experience on choice-inertia was greater when switching costs were high, while the interaction between EV gap and switching cost indicated that the (marginal) positive effect of EV gap on choice-inertia was reduced as switching costs increased. Lastly, the three-way interaction suggests a synergistic effect of choice number, EV gap, and switching costs on decreasing choice-inertia. Notably, switching costs appear to be the biggest contributor to choice-inertia, which likely masks the impact of choice number and EV gap on choice-inertia (e.g., the apparent ceiling effects in Fig 1).

**Table 1:** Logistic regression predicting choice-inertia in Study 1.

Predictor	*OR*^*a*^	*SE*^*b*^	*z*	*p*	CI 95%
Choice Number	1.06	.01	8.37	< .001	[1.04, 1.07]
EV Gap	1.21	.23	1.82	.07	[.99, 1.47]
Cost Condition	5.04	.79	10.27	< .001	[3.70, 6.86]
Choice Number X EV Gap	.99	.01	-.21	.83	[.99, 1.01]
Choice Number X Cost Condition	1.02	.01	3.02	.003	[1.01, 1.04]
EV Gap X Cost Condition	.63	.08	-3.63	< .001	[.49, .81]
Choice Number X EV Gap X Cost Condition	.98	.01	-2.04	.04	[.97, .99]
Constant	21.01	2.66	-24.03	< .001	[16.39, 26.93]

^a^Odds Ratio (*OR*)

^b^Robust Standard Error (*SE*).

#### EV maximization (Study 1)

**Fig 2:**
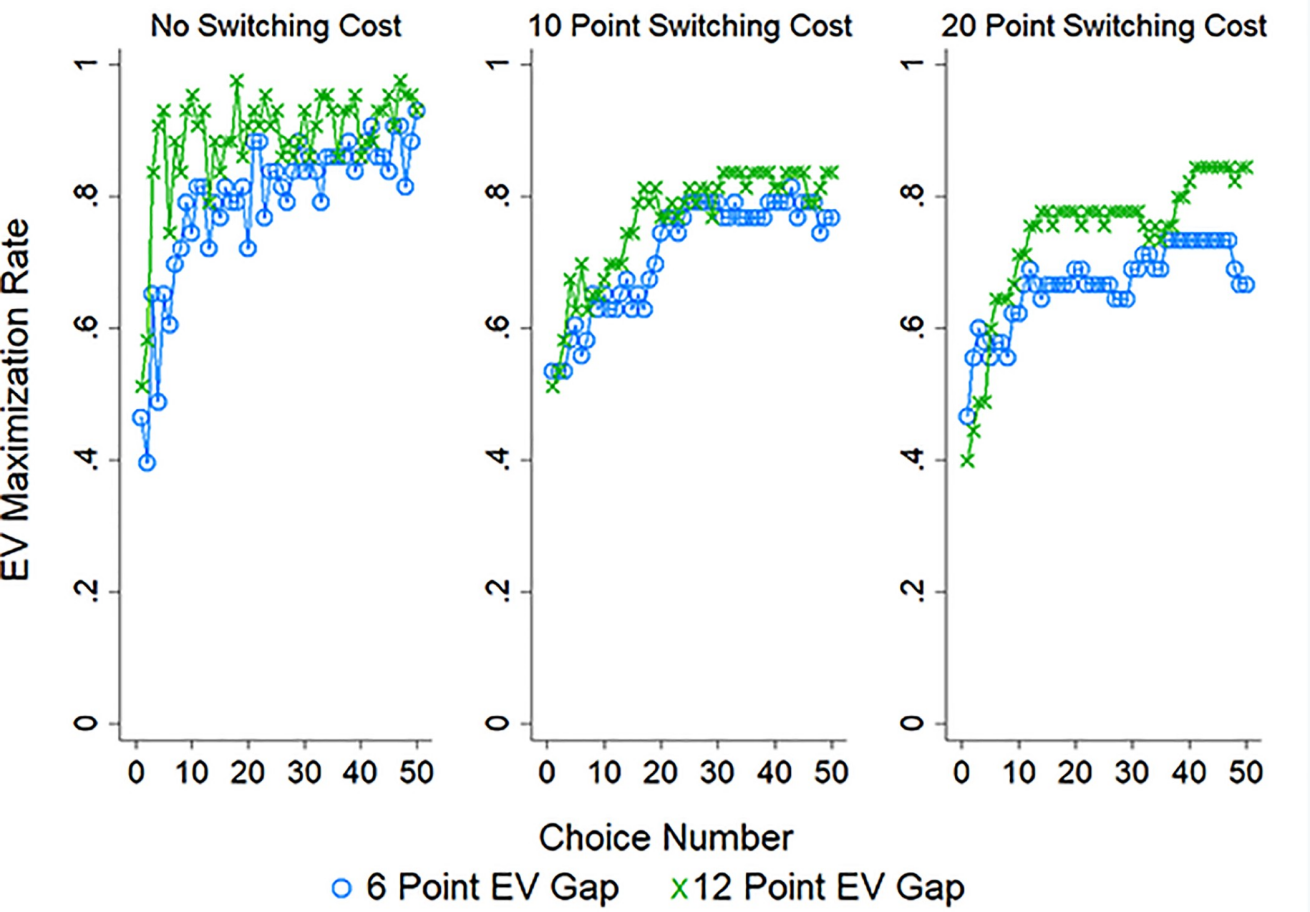
EV maximization in Study 1. The rate of EV maximization (choosing the option paying out a larger amount on average) in Study 1 with separate lines for each EV gap condition (pair of options with 0, 6, or 12 point gap in EV) and plotted separately by switching cost condition: 0 points (left panel), 10 points (middle panel), or 20 points (right panel).

Fig 2 displays the rate of EV maximization (choosing the option with a higher EV) across the 50 choices with separate lines for each EV gap condition (for the two pairs of options with disparate EVs), plotted separately by whether participants were in the 0 (left panel), 10 (middle panel), or 20 (right panel) point switching cost condition. Visual inspection suggests three patterns: EV maximization increased with experience and when the EV gap was larger, while EV maximization waned as switching costs increased.

As with choice-inertia we performed a logistic regression predicting EV maximization by choice number, EV gap, cost condition, as well as their interactions. The results of this analysis are reported in Table 2. In line with our observations the likelihood of EV maximization increased significantly over choices and as the EV gap between options increased, but decreased as the cost associated with switching increased. In addition, the increased likelihood of EV maximization over choices was diminished as the costs associated with switching increased. In sum, participants were sensitive to the difference in payouts provided by the options and the cost of switching, with the former leading to increased EV maximization, and the latter leading to decreased EV maximization.

**Table 2:** Logistic regression predicting EV maximization in Study 1.

Predictor	*OR*^*a*^	*SE*^*b*^	*z*	*p*	CI 95%
Choice Number	1.03	.01	6.33	< .001	[1.02, 1.03]
EV Gap	1.57	.26	2.69	.01	[1.13, 2.17]
Cost Condition	.69	.11	-2.18	.03	[.51, .96]
Choice Number × EV Gap	1	.01	.61	.54	[.99, 1.01]
Choice Number × Cost Condition	.99	.01	-2.59	.01	[.98, .99]
EV Gap × Cost Condition	.89	.15	-.67	.50	[.65, 1.24]
Choice Number × EV Gap × Cost Condition	1.01	.01	2.28	.02	[1, 1.02]
Constant	2.79	.41	7.01	< .001	[2.09, 3.71]

^a^Odds Ratio (*OR*)

^b^Robust Standard Error (*SE*).

#### The Relationship between choice-inertia and EV maximization in later choices (Study 1)

**Fig 3:**
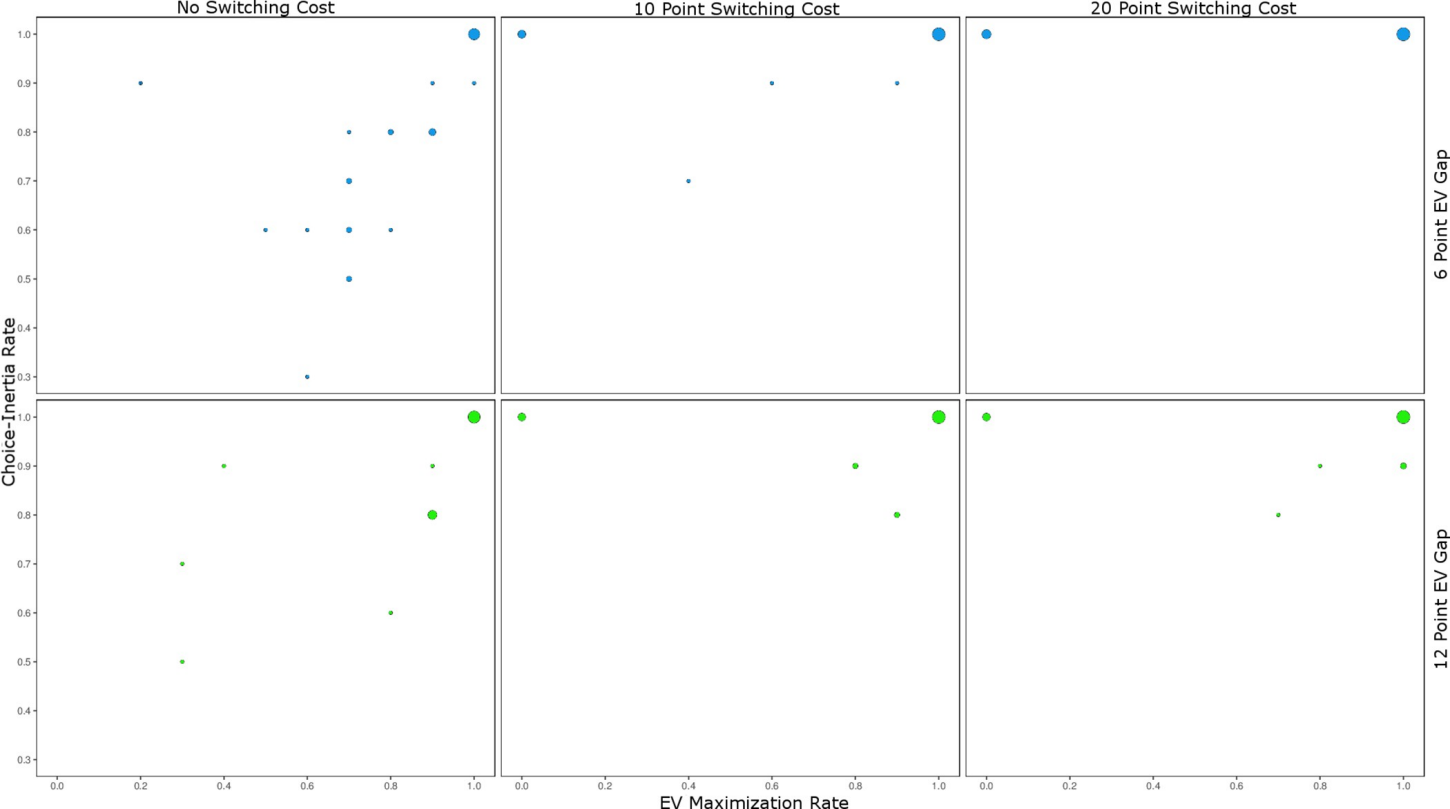
EV maximization by choice-inertia in Study 1. Bubble chart plotting EV maximization rate by choice-inertia rate in choices 38 to 47 plotted separately by EV gap condition (6 and 12 point differences in EV in the top and bottom panels, respectively) and switching cost condition: 0 points (left panels), 10 points (middle panels), and 20 points (right panels) in Study 1. The sizes of the bubbles indicate the number of participants at that intersection.

While the previous analyses provide some insight into choice-inertia and EV maximization, they provide only indirect insight into whether choice-inertia played a positive or negative role in participants EV maximization. To more directly assess the direction of choice-inertia (i.e., more or less EV maximization) we examined the rate of EV maximization in choices 38–47 (i.e., how many out of 10 choices a participant picked the EV maximizing option). We examine this range of choices instead of the last 10 choices because in the chosen range for all cost conditions switching from the smaller outcome option to the larger outcome option would on average lead to a net benefit (i.e., earning more from the subsequent EV maximizing choices than the switching cost). Fig 3 plots the rate of EV maximization by the choice-inertia rate in these 10 choices: Plotted separately by EV gap (6 point EV gap top panels; 12 point EV gap bottom panels) and switching cost condition (left panels 0 cost; middle panels 10 cost; right panels 20 cost). Visual inspection suggests two potential effects: A larger EV gap and larger switching costs appear to be associated with increased choice-inertia for both the inferior and EV maximizing options.

We performed a logistic regression predicting whether participants who showed 100% choice consistency (213 cases out of 262) never selected the EV maximizing option (coded 0: 34 observations) or always did (coded 1: 179 observations) by EV gap, cost condition, and their interaction. Table 3 presents this analysis. Only the cost associated with making a switch was predictive, and it predicted an overall decrease in EV maximization: 100% EV maximization with no switching cost, 81% EV maximization with a 10 switching cost, and 78% EV maximization when the cost of a switch was 20. Nevertheless, EV maximization across all participants (top right bubbles in Fig 3) did appear to increase with switching costs as well: 58%, 74%, and 77% of all participants showed 100% EV maximization across the no, 10, and 20 switching cost conditions, respectively. Thus, switching costs increased the proportion of participants who show 100% choice consistency, increasing EV maximization for some, but not for others.

**Table 3:** Logistic regression predicting whether participants exhibiting 100% choice consistency always or never EV maximized in choices 38–47 in Study 1.

Predictor	*OR*^*a*^	*SE*^*b*^	*z*	*p*	CI 95%
EV Gap	1.36	.39	1.04	.29	[.76, 2.41]
Cost Condition	.42	.12	-3.07	.002	[.24, .73]
EV Gap × Cost Condition	1.19	.35	.60	.55	[.67, 2.11]
Constant	6.22	1.56	7.29	< .001	[3.80, 10.17]

^a^Odds Ratio (*OR*)

^b^Robust Standard Error (*SE*).

### Study 1 discussion

In line with our predictions we find that experience (i.e., repeated choice), option similarity (i.e., the EV gap), and switching costs all influenced the level of choice-inertia observed. With more experience the rate of choice-inertia increased, presumably because decision makers learned which option they preferred. Increasing the EV gap between options led to increased choice-inertia as well, likely because it made it easier to identify the superior option: An interpretation supported by the finding that increasing the EV gap also increased the likelihood of EV maximization. Nevertheless, the cost of making a switch appeared to have the largest immediate impact with the rate of choice-inertia being greater when switching costs were (present) higher. In terms of the consequences of choice-inertia, only larger switching costs were predictive of decreased EV maximization. At the same time, the proportion of participants consistently showing EV maximization increased with switching cost. Thus, it appears that increasing the costs of exploration increases the proportion of individuals showing 100% choice-inertia, which in many cases (e.g., where limited exploration leads to an inferior option being identified as superior) led to continued exploitation of inferior options (i.e., inertia bias), but this did not reduce overall rates of choice-inertia which resulted in EV maximization.

## Study 2: Inertia in dynamic environments

In Study 2 we examine whether the effects found in Study 1 are replicated in a dynamic environment. As noted, several important behavioral problems like the sunk cost effect and learned helplessness can be described as sticking to an alternative which is good at first but becomes inferior at some point in time. Poor performance following choice-inertia in this context can result from obliviousness to the change (i.e., being able to detect the change) or to the potential benefits of switching (e.g., the increased earnings a switch would provide). In the current study environmental change was implemented by swapping the two options so that the superior option become inferior and vice versa. Will participants adapt to the change? We expect this to be dependent on the degree to which the change can be detected and the expected benefits from switching after the occurrence of the change. For example, if two options are relatively similar in their payouts (a smaller EV gap), swapping the two options becomes less salient than in the case of two distant options (options with large differences in EV), and the benefit associated with switching back to the superior alternative becomes smaller. In addition, when the gap is large, replacement can cause a stronger surprise (e.g., participants get used to high values and then suddenly they observe a low value) which by itself can lead to inertia offset (known as Surprise-Triggers-Change [27]). Furthermore, if the cost of a switch is high, the perceived benefit of making the switch to the higher value option might be reduced or in some cases completely ignored. For example, a decision maker who is considering switching to an option that returns 6 points more on average than the option they are currently choosing, but must pay 20 points to make the switch, must calculate the expected return four choices into the future to understand the switch as beneficial (i.e., that the switching cost of 20 points is lower than the net benefit returned). That is, the decision maker needs to expend more cognitive effort to see the value in making a switch. We therefore expect to observe more choice-inertia for the inferior option as the similarity of the options and switching costs increase.

### Methods

#### Participants

Two-hundred and fifty-three (*M*_age_ = 39.43; 56% female, 43% male, and 1% other) new participants were recruited using Amazon Mechanical Turk. Participants received $0.50 plus monetary incentives (*M* = $0.51) based on their decisions (described below). The same attention check used in Study 1 was employed and as in Study 1 participants (N ≈ 210) who failed it were not allowed to take part.

#### Stimuli and procedure

Study 2 was identical to Study 1 except for three changes: Only the two pairs with an EV maximizing option were included; participants only made choices in one randomly assigned pair (that is, in this study option similarity was manipulated between participants); instead of 50 choices 100 choices were made and after the 50^th^ choice the options were reassigned to the other label (e.g., the option assigned to Option A was reassigned to Option B and vice versa). Thus, participants were randomly assigned to an EV gap and a switching cost condition, switching cost of 0 (N = 37), 10 (N = 40), 20 (N = 43), then made 100 choices with the options being reassigned to labels on choice 51 (i.e., the maximizing option became the inferior option).

### Study 2 results

#### The development of choice-inertia with experience (Study 2)

**Fig 4:**
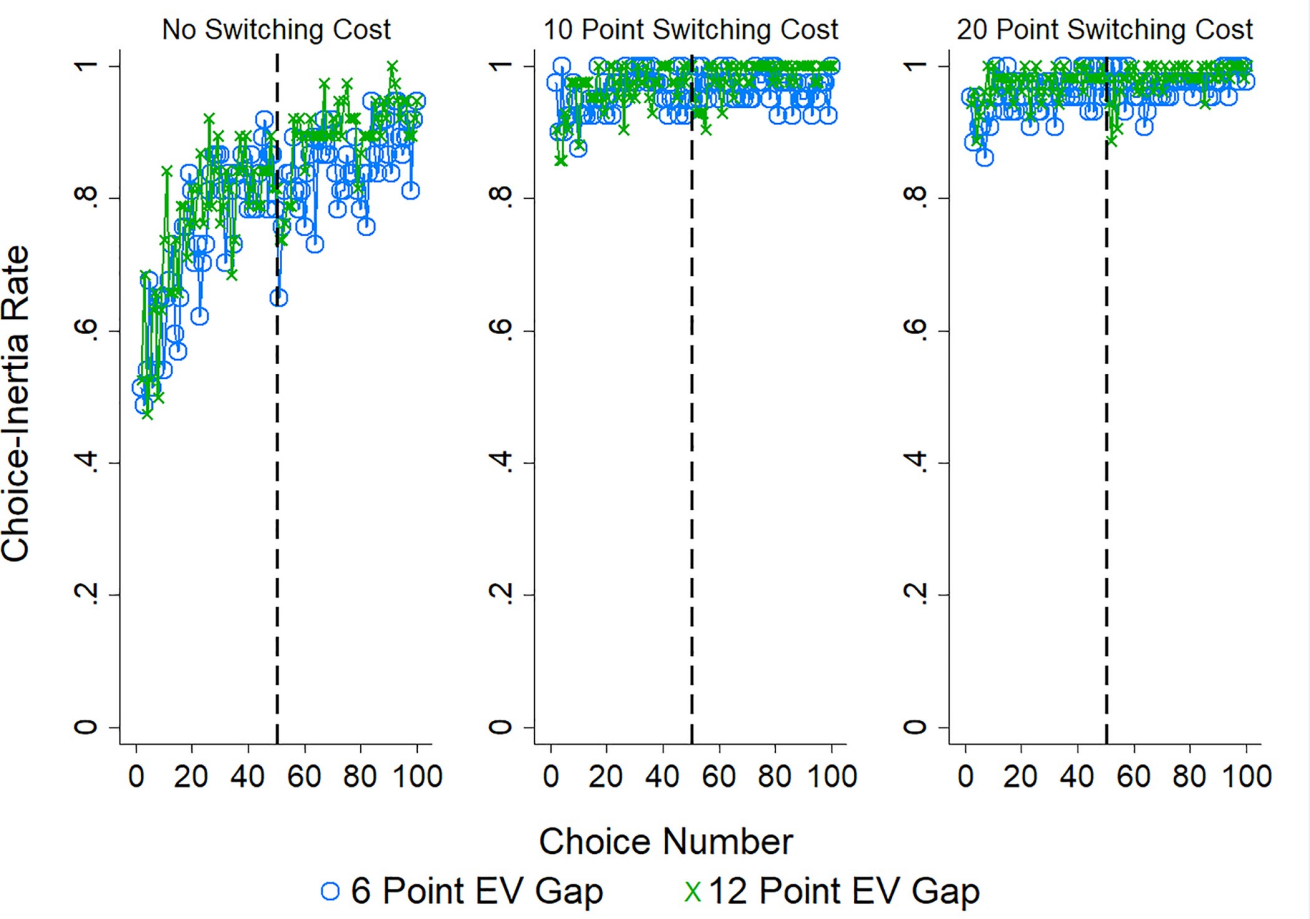
Choice inertia in Study 2. The rate of choice-inertia (choosing the same option on consecutive choices) in Study 2 with separate lines for EV gap condition (pairs of options with a 6 or 12 point differences in EV) and plotted separately by switching cost condition: 0 points (left panel), 10 points (middle panel), or 20 points (right panel). Horizontal dotted line indicates the point where the options labels were changed (after choice 50).

Fig 4 displays the rate of choice-inertia (choosing the same option on consecutive choices) across the 100 choices with separate lines for each EV gap condition and plotted separately by whether participants were in the 0 (left panel), 10 (middle panel), or 20 (right panel) point switching cost condition. Visual inspection appears to suggest three effects present in Study 1 are likely present in Study 2: Choice-inertia increased with experience, as the EV gap increased, and when there was a cost associated with making a switch. In addition, there appears to be a slight decrease in choice-inertia following the change in option label assignment which is most pronounced when there was no switching cost.

As in Study 1 we performed a logistic regression predicting choice-inertia by choice number, EV gap, cost condition, a dummy variable indicating whether the choice was before or after the change in option label assignment, as well as their interactions. The results of this analysis are reported in Table 4. In line with our observations we find the likelihood of choice-inertia increased with experience and as switching costs increased. The change in label assignment (Half), on the other hand, resulted in decreased choice-inertia. In addition, while the main effect of EV gap was not significant, the effect of increased choice-inertia with experience was greater as the gap grew larger, an effect that was amplified following the change in label assignment. Thus, like Study 1, choice-inertia increased with experience and when switching costs were high. And, while there was a decrease in choice-inertia following the change in option labeling, choice-inertia increased with experience to a greater extent when the EV gap was larger.

**Table 4:** Logistic regression predicting choice-inertia in Study 2.

Predictor	*OR*^*a*^	*SE*^*b*^	*z*	*p*	CI 95%
Choice Number	1.03	.01	5.73	< .001	[1.02, 1.04]
EV Gap	1.18	.38	.51	.61	[.63, 2.21]
Cost Condition	3.11	.59	5.95	< .001	[2.14, 4.52]
Half	.44	.09	-3.90	< .001	[.29, .66]
Choice Number × EV Gap	1.02	.01	2.18	.03	[1, 1.04]
Choice Number × Cost Condition	1	.01	.53	.59	[.99, 1]
Choice Number × Half	.99	.01	-.20	.84	[.99, 1.01]
EV Gap × Cost Condition	1.04	.39	.11	.92	[.49, 2.19]
EV Gap × Half	.49	.20	-1.74	.08	[.22, 1.09]
Cost Condition × Half	.72	.17	-1.38	.17	[.45, 1.15]
Choice Number × EV Gap × Cost Condition	1.02	.01	1.52	.13	[.99, 1.04]
Choice Number × EV Gap × Half	1.03	.01	2.70	.007	[1.01, 1.05]
Choice Number × Cost Condition × Half	1.01	.01	1.34	.18	[.99, 1.02]
EV Gap × Cost Condition × Half	.41	.19	-1.91	.06	[.16, 1.02]
Choice Number × EV Gap × Cost Condition × Half	1.01	.01	1.06	.29	[.99, 1.04]
Constant	21.19	3.41	18.98	< .001	[15.46, 29.05]

^a^Odds Ratio (*OR*)

^b^Robust Standard Error (*SE*).

#### EV maximization (Study 2)

**Fig 5:**
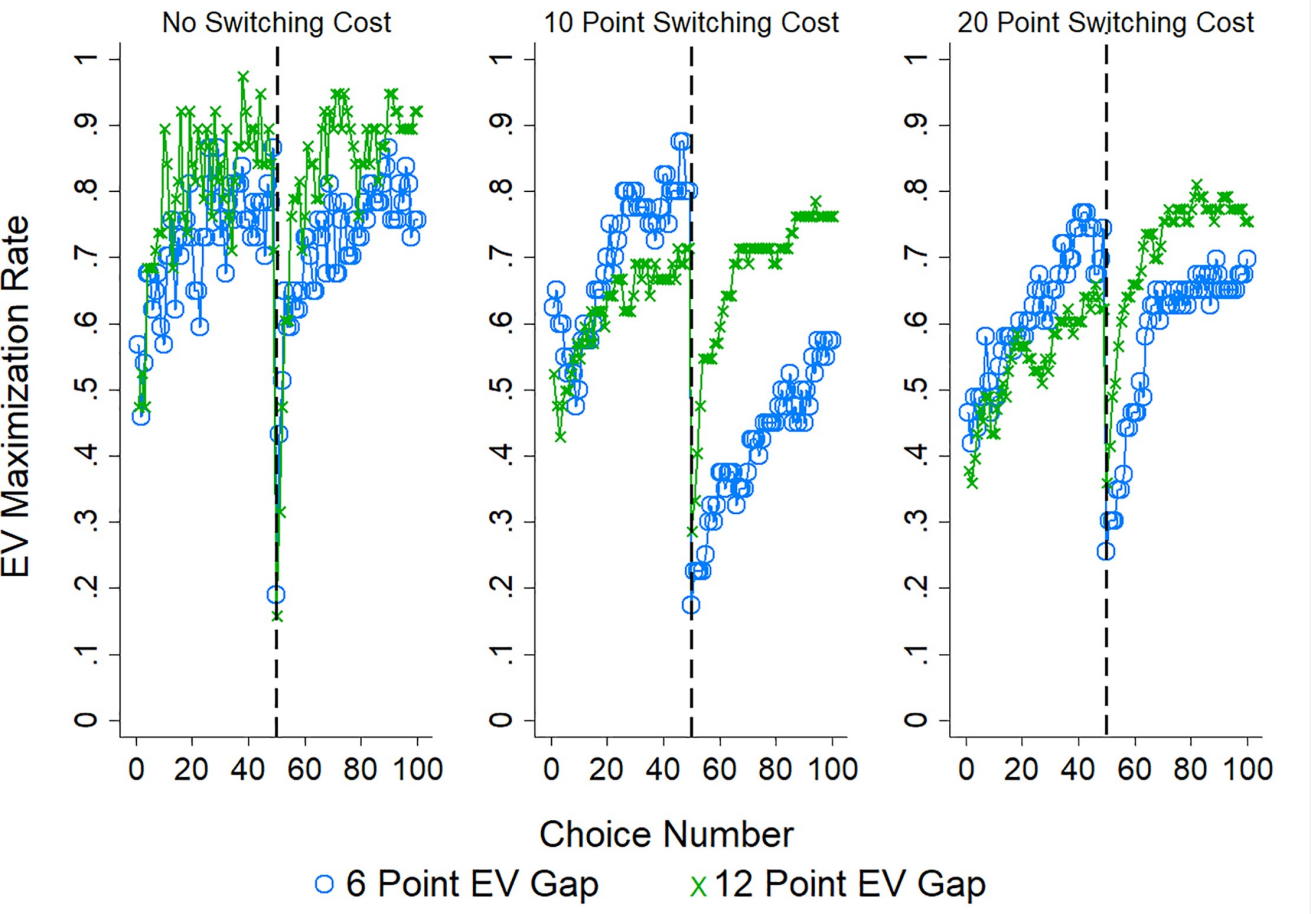
EV maximization in Study 2. The rate of EV maximization (choosing the option paying out a larger amount on average) in Study 2 with separate lines for each EV gap condition (pair of options with 0, 6, or 12 point gap in EV) and separate panels for each switching cost condition: 0 points (left panel), 10 points (middle panel), or 20 points (right panel). Horizontal dotted line indicates the point where the options labels were changed (after choice 50).

Fig 5 displays the rate of EV maximization across the 100 choices with separate lines for EV gap condition and plotted separately by whether participants were in the 0 (left panel), 10 (middle panel), or 20 (right panel) point switching cost condition. Visual inspection appears to suggest two effects present in Study 1 are present in Study 2: EV maximization increases with experience and is lower when switching costs were higher. In addition, while there is an initial decrease following the change, EV maximization increased over the remaining 50 choices; more so when the EV gap was larger and when switching costs were absent.

As with choice-inertia we performed a logistic regression predicting EV maximization by choice number, EV gap, cost condition, whether the decision was made before or after the change in option labels, as well as their interactions. The results are reported in Table 5. In line with our observations we find the likelihood of EV maximization increased with experience and when the EV gap was larger, while EV maximization decreased when costs were higher and following the change in options labels (Half). Nevertheless, following the change in option labels experience led to a recovery in the likelihood of EV maximization, and when the EV gap was larger the changes negative effect on EV maximization was diminished.

**Table 5:** Logistic regression predicting EV maximization in Study 2.

Predictor	*OR*^*a*^	*SE*^*b*^	*z*	*p*	CI 95%
Choice Number	1.02	.01	10.41	< .001	[1.02, 1.03]
EV Gap	1.31	.16	2.21	.03	[1.03, 1.66]
Cost Condition	.74	.05	-4.19	< .001	[.64, .85]
Half	.35	.06	-5.80	< .001	[.25, .50]
Choice Number × EV Gap	.99	.01	-.02	.98	[.99, 1.01]
Choice Number × Cost Condition	1	.01	.04	.97	[.99, 1]
Choice Number × Half	1.01	.01	2.63	.01	[1, 1.02]
EV Gap × Cost Condition	.91	.13	-.64	.52	[.68, 1.21]
EV Gap × Half	2.55	.92	2.60	.01	[1.26, 5.17]
Cost Condition × Half	1.11	.22	.53	.59	[.75, 1.64]
Choice Number × EV Gap × Cost Condition	.99	.01	-1.43	.15	[.98, 1.02]
Choice Number × EV Gap × Half	1.01	.01	1.09	.28	[.99, 1.02]
Choice Number × Cost Condition × Half	.99	.01	-.77	.44	[.99, 1.01]
EV Gap × Cost Condition × Half	1.88	.75	1.57	.12	[.86, 4.11]
Choice Number × EV Gap × Cost Condition × Half	.99	.01	-.79	.43	[.98, 1.01]
Constant	1.79	.11	9.67	< .001	[1.59, 2.02]

^a^Odds Ratio (*OR*)

^b^Robust Standard Error (*SE*).

#### The Relationship between choice-inertia and EV maximization in later choices (Study 2)

**Fig 6:**
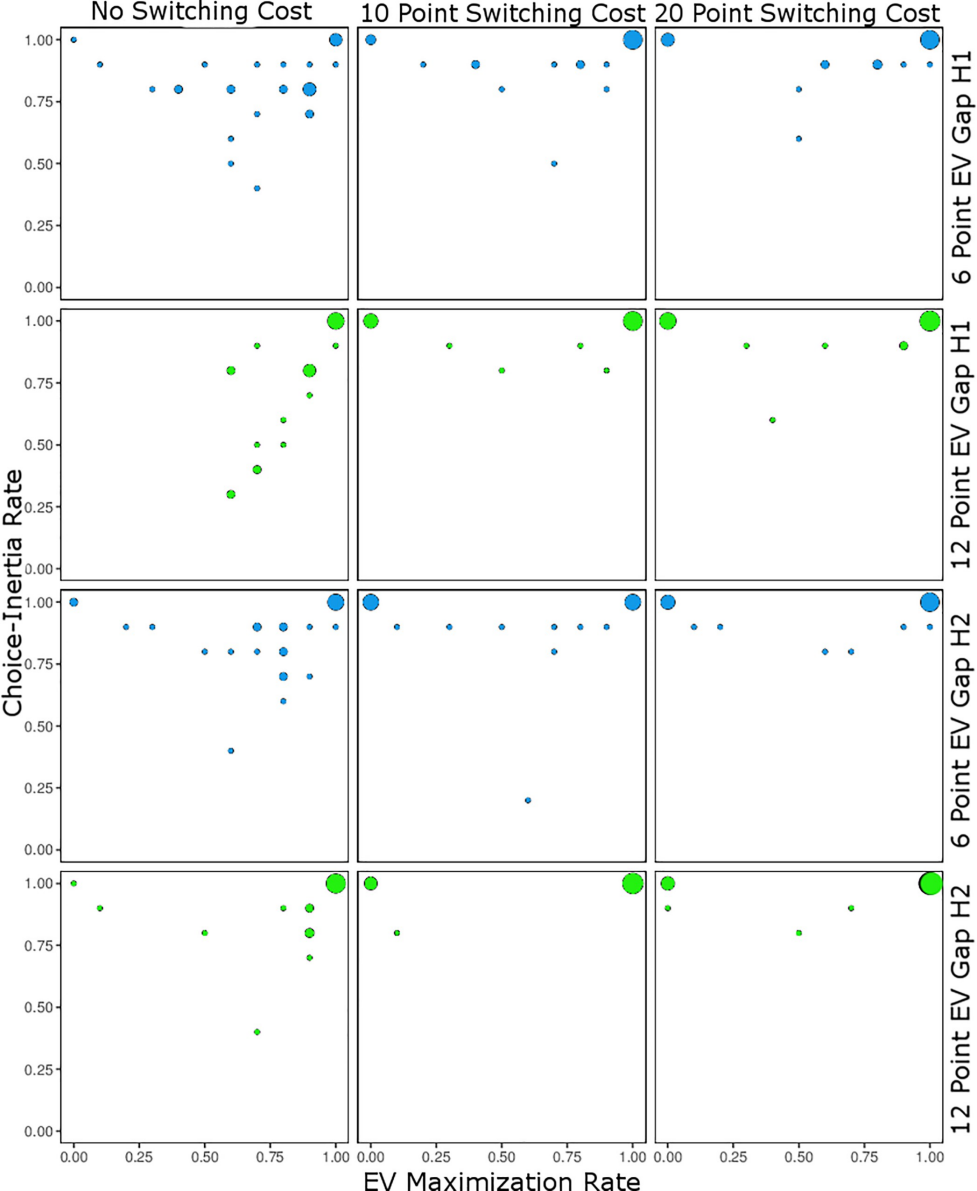
EV maximization by choice-inertia in Study 2. Bubble chart plotting EV maximization rate by choice-inertia rate in choices 38 to 47 and 88 to 97 plotted separately by EV gap condition (6 and 12 point differences in EV in rows one and three and two and four, respectively), switching cost condition (0 points left panels, 10 points middle panels, and 20 points right panels), and change in option labels (top panels before the change, bottom panels after the change) in Study 2. The sizes of the bubbles indicate the number of participants at that intersection.

As in Study 1 we examined the effect of choice-inertia on EV maximization in choices 38–47 and 88–97. As before, these ranges were employed because in all cost conditions switching from the smaller EV option to the EV maximizing option would on average lead to a net benefit. Fig 6 plots the rate of EV maximization by the choice-inertia rate in these 10 choices: Plotted separately by EV gap condition (6 point EV difference first and third row of panels; 12 point EV difference second and fourth row of panels), cost condition (left panels 0 cost; middle panels 10 cost; right panels 20 cost), and by change in option labels (top panels choices 38–47; bottom panels choices 88–97). Visual inspection suggests two effects present in Study 1 might appear in Study 2: When the EV gap was larger, or there was a larger cost associated with a switch, there was more choice-inertia, for some leading to EV maximization and for others not. There also appears to be a small effect of whether the choices were made before or after the change in option labels with more inertia following the change than preceding it.

As in Study 1 we performed a logistic regression predicting whether participants who showed 100% choice consistency (384 out of 506 observations) never (coded 0: 94 observations) or always EV maximized (coded 1: 290 observations) by EV gap, cost condition, change in option labels (half), and their interaction. The results of this analysis are presented in Table 6. As in Study 1 increasing switching costs predicted a decrease in EV maximization: 95% EV maximization with no switching cost, 71% EV maximization with a 10 switching cost, and 71% EV maximization when the cost of a switching was 20. Nevertheless, the proportion of all participants (pooled across half) showing EV maximization also increased as switching costs increased: 48%, 61%, and 63%, respectively. In addition, following the change in option labels when the EV gap was larger EV maximization was more likely. Thus, as in Study 1 when switching costs were higher participants displayed more choice-inertia for the inferior option. And, there was an increase in the likelihood of EV maximization following a change in the environment when the EV gap was larger.

**Table 6:** Logistic regression predicting whether participants exhibiting 100% choice consistency always or never EV maximized in choices 38–47 and 88–97 in Study 2.

Predictor	OR^a^	SE^b^	*z*	*p*	CI 95%
EV Gap	1.32	.29	1.26	.21	[.85, 2.05]
Cost Condition	.54	.08	-4.19	< .001	[.39, .72]
Half	.96	.28	-.14	.89	[.54, 1.71]
EV Gap × Cost Condition	.79	.24	-.75	.45	[.43, 14.40]
EV Gap × Half	4.47	2.67	2.51	.01	[1.39, 15.51]
Cost Condition × Half	1.42	.53	.94	.35	[.68, 2.93]
EV Gap × Cost Condition × Half	1.02	.79	.03	.98	[.22, 4.71]
Constant	3.45	.39	11.08	< .001	[2.77, 4.29]

^a^Odds Ratio (*OR*)

^b^Robust Standard Error (*SE*).

### Study 2 discussion

In Study 2 we replicate the general pattern of findings observed in Study 1: Increased EV maximization with experience which was amplified when there was a larger difference in the options EVs; increased choice-inertia (primarily for the inferior option) as the switching cost increased. In terms of the effect of the options changing labels we find that choice-inertia decreased following the change, more so when making a switch was associated with a higher cost, as did EV maximization. The change in labels did not appear to have any robust effect on the consequences of inertia, though we did find more choice-inertia of the EV maximizing option following the change when there was a greater difference in the options EVs. Thus, as in Study 1, the most robust predictor of choice-inertia appears to be switching costs.

## General discussion

In the current studies we sought to gain insight into what features of the environment lead to choice-inertia and its consequences. Specifically, we provided a direct test of whether switching costs increase choice-inertia, and whether that increase in choice-inertia is more likely to be directed towards or away from EV maximizing options. In both studies increasing switching costs increased choice-inertia, decreased overall EV maximization, and appeared to dampen the effects of experience and EV gap on choice-inertia and EV maximization. Thus, in line with our predictions we provide direct evidence that switching costs increase choice-inertia, and can bias the direction of that inertia.

Why did switching costs increase choice-inertia for inferior options? One possibility is that the increased switching costs led to less thorough initial exploration. For example, instead of searching each option three times a large switching cost might have led participants to search each option less, skewing their perceptions of the options values. Similarly, increasing the cost of making a switch may have led participants to employ search strategies they did not favor. For example, although summary search which involve less switching was found to increase maximization [24] this was the case only for participants who employed this strategy naturally. It could be that for others who employed this search strategy only due to the increased switching cost, cognitive demand increased [8], leading to miscalculations in the difference in the options values. Alternatively, participants may have gained accurate representations of the options values, but been unable (unmotivated) to perform the calculations required to realize that making a switch although potentially costly in the short term, would be beneficial in the long term. One direction for future research then is attempting to understand how explorative costs bias the consequences of choice-inertia. By understanding what is at the root of this bias a successful debiasing intervention might be developed.

However, it is important to note that although EV maximization is a popular measure, it does not necessarily imply optimality, because choice-optimality can be argued to include a plethora of other variables such as metabolic costs, mental effort, and the value of time. Also, as mentioned in [28] and [29], other ecologically plausible tradeoffs such as the “speed value tradeoff” allude to the fact that high vs. low value environments can induce different optimal strategies. As a first step into this bigger picture we examined decision times (DT) for the different conditions and we provide the data and figures in the S1 Appendix. To summarize, the DT analysis shows that in later trials higher switching costs led to shorter decision times (an average gap of about 150 ms per choice toward the end of the task). This result is in line with the finding that switching cost increased overall choice-inertia, which in turn saves time (in fact, the gap between participants who exhibited 100% choice-inertia in last trials and participants who did not is around 150ms per choice as well). If the time saved is sufficiently large and differences in rewards are sufficiently low, choice inertia of the inferior option could then be considered optimal. In the current setting choice-inertia could save a few seconds, yet reward differences were also small, thus optimality remains ambiguous. In addition, the current setting can be viewed as a simplified 2-armed bandit problem with switching costs, where the definition of optimal solution might not exist [30] [31]. Thus, our findings mainly refer to EV maximization (rather than optimality) and show that switching costs increase choice inertia of selecting the inferior option, which harms EV maximization.

As outlined in the introduction understanding choice-inertia is important because it is theorized to underly/reflect many biases affecting decision making. Sunk cost effects [2] for example, where decision makers continue to invest in projects (follow courses of action) which are no longer beneficial fit squarely with adverse consequences of choice-inertia. Interestingly, recent research indicates that sunk costs effects can even be transferred from person-to-person [32], suggesting an interesting possibility that choice-inertia might be contagious. In the current studies we found that choice-inertia for selecting inferior options increased when switching costs increased. Similarly, the switching costs have also been suggested to influence adverse choice-inertia in resistance to information sharing via management systems [33], when deciding on changing healthcare plans [34], and the failure of cryptocurrencies to become a serious competitor for traditional fiat currencies [35]. The similar impact of switching costs in each of these contexts suggests a likely relationship between the underlying factors leading to choice-inertia in each, highlighting a potentially fruitful line of research: Investigating whether choice-inertia in these varied decision contexts develops (manifests) similarly within an individual. For example, are individuals who show increased choice-inertia in repeated choice contexts, like those studied here, also likely to show larger sunk costs? Be more resistant to organizational change? More likely to imitate the inertia of others? Or, more likely to be influenced by default effects? Establishing the presence (or absence) of such a relationship would help bridge somewhat disparate theoretical accounts for these behavioral oddities. In addition, it could lead to the development of an individual difference measure capturing individual susceptibility to inertia related effects, providing identification of those decision makers who would benefit most from intervention (e.g., a decision support system).

In short, the current studies provided a novel direct investigation into the effects of switching costs on choice-inertia and indicated that larger switching costs increased choice-inertia mainly associated with sticking to inferior options. Providing initial empirical support for the proposition that switching costs influence choice-inertia and clarify their influence by showing that for a growing proportion of decision makers larger switching costs led to choice-inertia with adverse consequences on earnings. As such, the current studies provide evidence that garnering a better understanding of choice-inertia should be of central importance to decision scientists given its potential to hamper effective decision making across a variety of contexts.

## Supporting information

S1 Appendix (DOCX)

## References

[1] Samuelson W, Zeckhauser R. Status quo bias in decision making. Journal of risk and uncertainty. 1988 3 1;1(1):7–59.

[2] Arkes HR, Blumer C. The psychology of sunk cost. Organizational behavior and human decision processes. 1985 2 1;35(1):124–40.

[3] Johnson EJ, Goldstein D. Do defaults save lives?. Science. 2003;302(5649):1338–1339. 10.1126/science.1091721 14631022

[4] Busemeyer JR, Swenson KN, Lazarte A. An adaptive approach to resource allocation. Organizational Behavior and Human Decision Processes. 1986 12 1;38(3): 318–41.

[5] Busemeyer JR, Myung IJ. Resource allocation decision making in an uncertain environment. Acta Psychologica. 1987 10 1;66(1):1–9.

[6] Teodorescu K, Erev I. On the decision to explore new alternatives: The coexistence of under‐and over‐exploration. Journal of Behavioral Decision Making. 2014 4;27(2):109–23.

[7] Yechiam E, Erev I, Gopher D. On the potential value and limitations of emphasis change and other exploration-enhancing training methods. Journal of Experimental Psychology: Applied. 2001 12;7(4):277 11838890

[8] Ashby NJ, Rakow T. Eyes on the prize? Evidence of diminishing attention to experienced and foregone outcomes in repeated experiential choice. Journal of Behavioral Decision Making. 2016 4 7;29(2–3):183–93.

[9] Denrell J, March JG. Adaptation as information restriction: The hot stove effect. Organization Science. 2001 10;12(5):523–38.

[10] Maier SF, Seligman ME. Learned helplessness: theory and evidence. Journal of experimental psychology: general. 1976 3;105(1):3.

[11] Teodorescu K, Erev I. Learned helplessness and learned prevalence: Exploring the causal relations among perceived controllability, reward prevalence, and exploration. Psychological science. 2014 10;25(10):1861–9. 10.1177/0956797614543022 25193942

[12] Kim HW, Kankanhalli A. Investigating user resistance to information systems implementation: A status quo bias perspective. MIS quarterly. 2009 9 1:567–82.

[13] Polites GL, Karahanna E. Shackled to the status quo: The inhibiting effects of incumbent system habit, switching costs, and inertia on new system acceptance. MIS quarterly. 2012 3 1;36(1).

[14] Erwin DG, Garman AN. Resistance to organizational change: linking research and practice. Leadership & Organization Development Journal. 2010 2 6;31(1):39–56.

[15] Gal D. A psychological law of inertia and the illusion of loss aversion. Judgment and Decision Making. 2006; 1(1): 23−32.

[16] Todd PM, Gigerenzer G. Environments that make us smart: Ecological rationality. Current directions in psychological science. 2007 6;16(3):167–71.

[17] Marshall JA, Favreau-Peigne A, Fromhage L, Mcnamara JM, Meah LF, Houston AI. Cross inhibition improves activity selection when switching incurs time costs. Current zoology. 2015 4 1;61(2):242–50.

[18] Dixit A. Investment and hysteresis. Journal of economic perspectives. 1992 3;6(1):107–32.

[19] Azar OH. The default heuristic in strategic decision making: When is it optimal to choose the default without investing in information search?. Journal of Business Research. 2014 8 1;67(8):1744–8.

[20] Dinner I, Johnson EJ, Goldstein DG, Liu K. Partitioning default effects: why people choose not to choose. Journal of Experimental Psychology: Applied. 2011 12;17(4):332 10.1037/a0024354 21707203

[21] Suri G, Sheppes G, Schwartz C, Gross JJ. Patient inertia and the status quo bias: when an inferior option is preferred. Psychological science. 2013 9;24(9):1763–9. 10.1177/0956797613479976 23873580

[22] Plonsky O, Teodorescu K, Erev I. Reliance on small samples, the wavy recency effect, and similarity-based learning. Psychological review. 2015 10;122(4):621 10.1037/a0039413 26075914

[23] Ashby NJ, Konstantinidis E, Yechiam E. Choice in experiential learning: True preferences or experimental artifacts?. Acta psychologica. 2017 3 1;174:59–67. 10.1016/j.actpsy.2017.01.010 28189706

[24] Hills TT, Hertwig R. Information search in decisions from experience: Do our patterns of sampling foreshadow our decisions?. Psychological science. 2010 12;21(12):1787–92. 10.1177/0956797610387443 20974711

[25] Rogers, 1991

[26] Barr DJ, Levy R, Scheepers C, Tily HJ. Random effects structure for confirmatory hypothesis testing: Keep it maximal. Journal of memory and language. 2013 4 1;68(3):255–78. 10.1016/j.jml.2012.11.001 PMC3881361 24403724

[27] Nevo I, Erev I. On surprise, change, and the effect of recent outcomes. Frontiers in psychology. 2012 2 21;3:24 10.3389/fpsyg.2012.00024 22363303 PMC3283116

[28] Teodorescu AR, Moran R, Usher M. Absolutely relative or relatively absolute: violations of value invariance in human decision making. Psychonomic bulletin & review. 2016 2 1;23(1):22–38. 26022836 10.3758/s13423-015-0858-8

[29] Pirrone A, Stafford T, Marshall JA. When natural selection should optimize speed-accuracy trade-offs. Frontiers in neuroscience. 2014 4 10;8:73 10.3389/fnins.2014.00073 24782703 PMC3989582

[30] Banks JS, Sundaram RK. Switching costs and the Gittins index. Econometrica. 1994;62(3):687–94.

[31] Jun T. A survey on the bandit problem with switching costs. De Economist. 2004 12 1;152(4):513–41.

[32] Olivola CY. The interpersonal sunk-cost effect. Psychological science. 2018 7;29(7):1072–83. 10.1177/0956797617752641 29750633

[33] Li J, Liu M, Liu X. Why do employees resist knowledge management systems? An empirical study from the status quo bias and inertia perspectives. Computers in Human Behavior. 2016 12 1;65:189–200.

[34] Duijmelinck DM, Mosca I, van de Ven WP. Switching benefits and costs in competitive health insurance markets: A conceptual framework and empirical evidence from the Netherlands. Health policy. 2015 5 1;119(5):664–71. 10.1016/j.healthpol.2014.11.015 25530069

[35] Luther WJ. Cryptocurrencies, network effects, and switching costs. Contemporary Economic Policy. 2016 7;34(3):553–71.

