# Supplementary material for: The effect of switching costs on choice-inertia and its consequences
Source: PLoS One. 2019 Mar 25;14(3):e0214098. doi: 10.1371/journal.pone.0214098 (PMC6433253; doi:10.1371/journal.pone.0214098)
Supplement: S1 Appendix — (DOCX) [file pone.0214098.s001.docx]

# Appendix – Decision times analyses:

## Study 1:


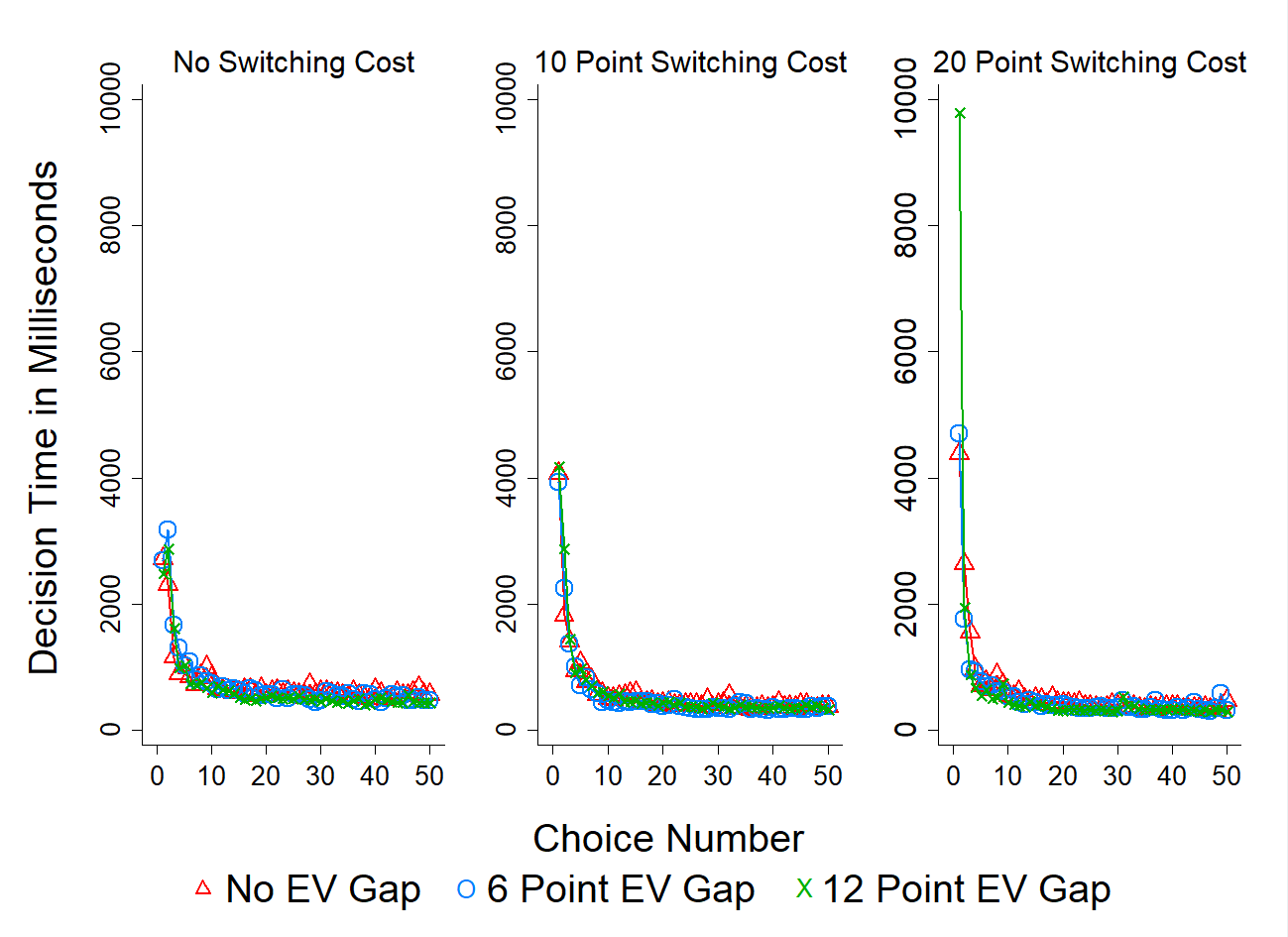
**S1 Fig. Average decision times in Study 1.**

Average decision time in millisecond (i.e., the time it took a participant to make a choice) in Study 1 with separate lines for each EV gap condition (pairs of options with a 0, 6, or 12 point EV gap) and plotted separately by switching cost condition: 0 points (left panel), 10 points (middle panel), or 20 points (right panel).

S1 Fig displays the decision times in milliseconds (i.e., the time it took a participant to make a choice) over choices plotted separately by EV Gap and Switching Cost conditions. It appears that in all conditions decision times reduce quickly with experience, though initial decisions appear to take longer when switching costs were higher. To directly investigate these inferences, we predicted decision times (skew corrected via log transformation) by choice number, EV gap, switching cost, as well as their interactions (see S1 Table). Decision times were found to decrease over choices and were shorter when switching costs were higher (*M_NoCos_*_t_ = 691.78; *M_10Cost_*  = 568.24; *M_20Cost_*  = 579.23). The interaction between choice number and switching costs indicate that the effect of choice number was larger when switching costs were high. The three-way interaction suggests that the synergistic effect of choice number and switching costs on decreasing decision times was slightly reduced when EV gaps were larger. In short, the current results align with those investigating inertia and suggest that with more experience less contemplation is made before each choice.

**S1 Table**. **Liner regression predicting (log transformed) decision times in Study 1**.

| **Predictor** | ***b*** | ***SE^a^*** | ***t*** | ***p*** | **CI 95%** |
| --- | --- | --- | --- | --- | --- |
| Choice Number | -.02 | .001 | -28.58 | < .001 | [-.02, -.01] |
| EV Gap | -.04 | .02 | -1.82 | .07 | [-.09, .01] |
| Cost Condition | -.22 | .06 | -3.93 | < .001 | [-.33, -.11] |
| Choice Number X EV Gap | -.001 | .001 | -.58 | .56 | [-.01, .01] |
| Choice Number X Cost Condition | -.002 | .001 | -2.44 | .02 | [-.01, -.001] |
| EV Gap X Cost Condition | .03 | .03 | -1.05 | .29 | [-.02, .08] |
| Choice Number X EV Gap X Cost Condition | .02 | .001 | 2.07 | .04 | [.001, .005] |
| Constant | 5.93 | .05 | 130.58 | < .001 | [5.84, 6.02] |

^a^Robust Standard Error (*SE*).

To determine whether decision times were related to the degree of choice-inertia and the direction of that inertia we compared the average decision times across choices 38-47 for those showing 100% choice-inertia for the EV maximizing option (*M* = 306.36), those showing 100% choice-inertia for the inferior option (*M* = 312.96), and those showing less than 100% consistency (*M* = 484.42) in a one-way ANOVA. A significant main effect was found, *F*(2,128) = 8.92, *p* <= .001. *Post-Hoc* comparison with Tukey adjustment indicated that while those who showed 100% choice-inertia for the EV maximizing option showed shorter decision times than those who did not show 100% choice-inertia (*p* < .001), they did not make faster decisions than those who showed 100% choice-inertia for the inferior option (*p* = .99); those not showing 100% choice inertia and those showing 100% choice-inertia for the inferior option did not differ significantly (*p* = .08).

## Study 2:


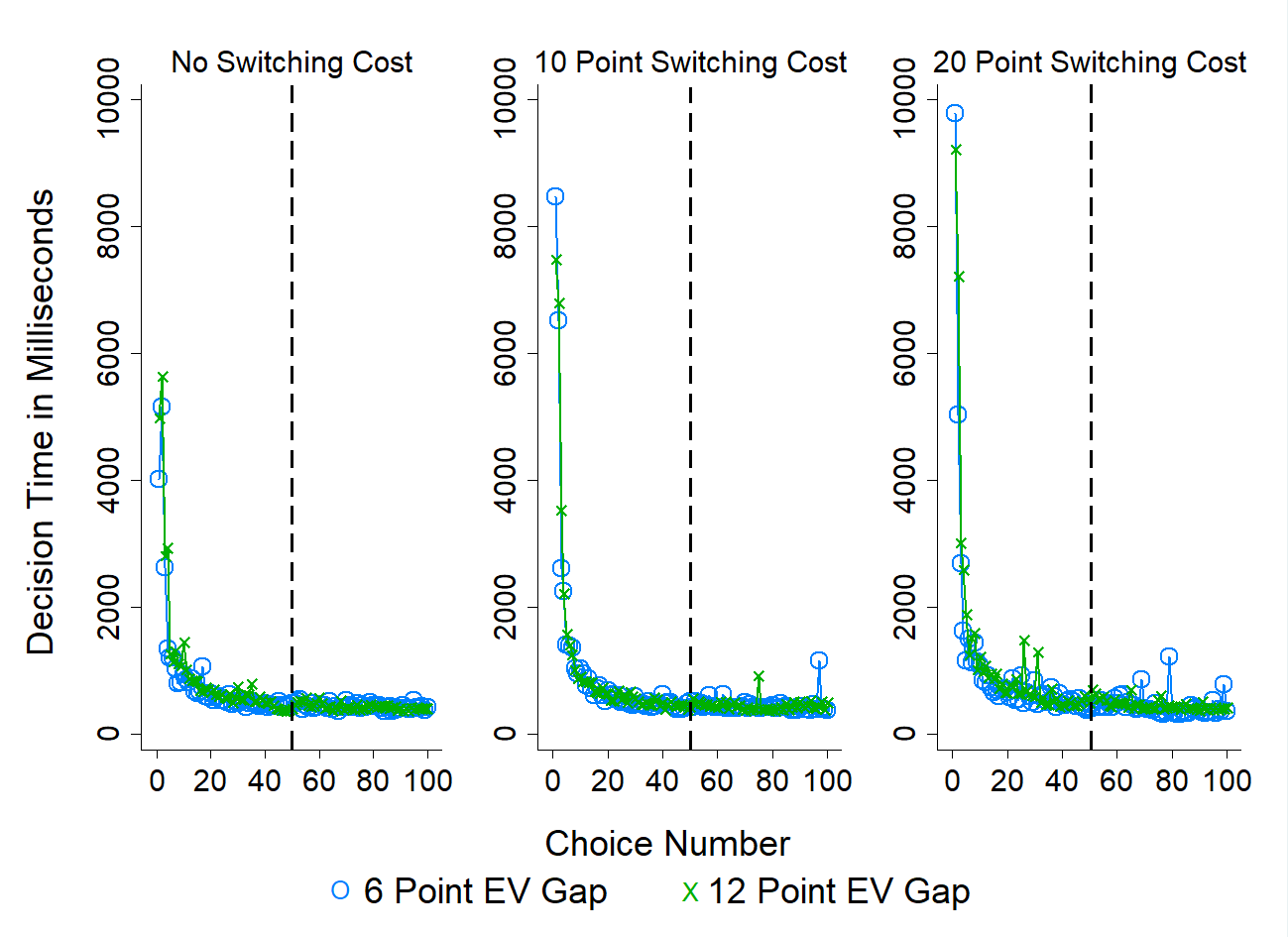


**S2 Fig. Average decision times in Study 2.**

Average decision time (i.e., the time it took a participant to make a choice) in Study 2 with separate lines for EV gap condition (pairs of options with a 6 or 12 point differences in EV) and plotted separately by switching cost condition: 0 points (left panel), 10 points (middle panel), or 20 points (right panel). Horizontal dotted line indicates the point where the options labels were changed (after choice 50).

S2 Fig displays the decision times over choices plotted separately by EV Gap and Switching Cost conditions. As in Study 1 it appears that in all condition’s decision times reduce with experience, though initial decisions again appear to take longer when switching costs were higher. There appears to be a small increase in decision times following the change in option labels as well. We predicted decision times (skew corrected via log transformation) by choice number, EV gap, switching cost, whether decision was made before or after the change in option labels, as well as their interactions (see S2 Table). As in Study 1 decision times decreased over choices. The interaction between choice number and switching costs indicates that the effect of choice number was larger when switching costs were higher as in Study 1. After the change in option labels decision times increased and the interaction with choice number suggests decision times decreased less with experience following the change. In sum, the current results replicate Study 1 showing that decision times decrease with experience and when the cost of switching is higher, and extend them by indicating that environmental changes can increase pre-choice considerations.

**S2 Table.** **Liner regression predicting (log transformed) decision times in Study 2.**

| **Predictor** | ***b*** | ***SE^a^*** | ***t*** | ***p*** | **CI 95%** |
| --- | --- | --- | --- | --- | --- |
| Choice Number | -.02 | .001 | -39.42 | < .001 | [-.019, -.017] |
| EV Gap | .02 | .08 | .33 | .74 | [-.12, .17] |
| Cost Condition | -.001 | .04 | -.02 | .99 | [-.09, .09] |
| Half | .43 | .03 | 20.09 | < .001 | [.39, .47] |
| Choice Number × EV Gap | -.001 | .001 | -1.22 | .23 | [-.003, .001] |
| Choice Number × Cost Condition | -.001 | .001 | -1.97 | .049 | [-.002, -.0001] |
| Choice Number × Half | .03 | .001 | 29.87 | < .001 | [.028, .03] |
| EV Gap × Cost Condition | .12 | .09 | 1.39 | .17 | [-.05, .29] |
| EV Gap × Half | .07 | .04 | 1.67 | .096 | [-.01, .15] |
| Cost Condition × Half | -.01 | .03 | -.25 | .81 | [-.06,.04] |
| Choice Number × EV Gap × Cost Condition | .001 | .001 | .70 | .48 | [-.001, .003] |
| Choice Number × EV Gap × Half | .002 | .002 | 1.09 | .28 | [-.002, .01] |
| Choice Number × Cost Condition × Half | -.001 | .001 | -.05 | .96 | [-.002, .002] |
| EV Gap × Cost Condition × Half | .001 | .05 | .01 | .99 | [.16, 1.02] |
| Choice Number × EV Gap × Cost Condition × Half | -.002 | .002 | -.97 | .34 | [-.007, .002] |
| Constant | 5.67 | .04 | 151.21 | < .001 | [5.60, 5.75] |

^a^Robust Standard Error (*SE*).
